# Efficient automated localization of ECoG electrodes in CT images via shape analysis

**DOI:** 10.1007/s11548-021-02325-0

**Published:** 2021-03-09

**Authors:** Jessica Centracchio, Antonio Sarno, Daniele Esposito, Emilio Andreozzi, Luigi Pavone, Giancarlo Di Gennaro, Marcello Bartolo, Vincenzo Esposito, Roberta Morace, Sara Casciato, Paolo Bifulco

**Affiliations:** 1 Department of Electrical Engineering and Information Technologies, Polytechnic and Basic Sciences School, University of Naples Federico II, Naples, Italy; 2 National Institute for Nuclear Physics (INFN), Naples, Italy; 3 Department of Neurorehabilitation, IRCCS Istituti Clinici Scientifici Maugeri, Pavia, Italy; 4 IRCCS Neuromed, Pozzilli, Italy; 5 Department of Human Neurosciences, Sapienza University, Rome, Italy

**Keywords:** Epilepsy surgery, ElectroCorticoGraphy, Electrodes recognition, CT image processing, Shape analysis, Gaussian Support Vector Machine

## Abstract

**Purpose:**

People with drug-refractory epilepsy are potential candidates for surgery. In many cases, epileptogenic zone localization requires intracranial investigations, e.g., via ElectroCorticoGraphy (ECoG), which uses subdural electrodes to map eloquent areas of large cortical regions. Precise electrodes localization on cortical surface is mandatory to delineate the seizure onset zone. Simple thresholding operations performed on patients’ computed tomography (CT) volumes recognize electrodes but also other metal objects (e.g., wires, stitches), which need to be manually removed. A new automated method based on shape analysis is proposed, which provides substantially improved performances in ECoG electrodes recognition.

**Methods:**

The proposed method was retrospectively tested on 24 CT volumes of subjects with drug-refractory focal epilepsy, presenting a large number (> 1700) of round platinum electrodes. After CT volume thresholding, six geometric features of voxel clusters (volume, symmetry axes lengths, circularity and cylinder similarity) were used to recognize the actual electrodes among all metal objects via a Gaussian support vector machine (G-SVM). The proposed method was further tested on seven CT volumes from a public repository. Simultaneous recognition of depth and ECoG electrodes was also investigated on three additional CT volumes, containing penetrating depth electrodes.

**Results:**

The G-SVM provided a 99.74% mean classification accuracy across all 24 single-patient datasets, as well as on the combined dataset. High accuracies were obtained also on the CT volumes from public repository (98.27% across all patients, 99.68% on combined dataset). An overall accuracy of 99.34% was achieved for the recognition of depth and ECoG electrodes.

**Conclusions:**

The proposed method accomplishes automated ECoG electrodes localization with unprecedented accuracy and can be easily implemented into existing software for preoperative analysis process. The preliminary yet surprisingly good results achieved for the simultaneous depth and ECoG electrodes recognition are encouraging.

Ethical approval n°NCT04479410 by “IRCCS Neuromed” (Pozzilli, Italy), 30th July 2020.

**Supplementary Information:**

The online version contains supplementary material available at 10.1007/s11548-021-02325-0.

## Introduction

Epilepsy affects 39 to 50 million people worldwide [1, 2], about 3–10 per 1000 [1]. Of these, 30–40% are drug-resistant and need alternative treatments [2]. Drug-refractory patients with focal epilepsy represent potential candidates to surgical treatment, which consists in the resection of the epileptogenic zone, defined as the site of the beginning of the epileptic seizures. A Cochrane review reported that 65% of about 16,000 patients had a good outcome from surgery [1], but it strongly depends on accurate localization of the seizure onset zone [3]. In about 70% of patients, the localization is achieved by combining neuroimaging techniques with noninvasive electrophysiological recordings, such as ElectroEncephaloGraphy (EEG) [1]. However, EEG does not provide very accurate location of the epileptogenic zone, and especially for drug-resistant epileptic patients, invasive electrophysiological investigations should be carried out. ElectroCorticoGraphy (ECoG) is the most widespread technique to acquire intracranial EEG and is performed by implanting subdural electrodes directly onto the patient's brain surface [4]. Compared to the EEG electrodes applied on the scalp, the subdural electrodes provide a signal with a much higher resolution and allow a very clear view of the small activity foci [5]. Subdural electrodes allow not only the localization of abnormal epileptic tissue, but also the localization of adjacent normal functions. Therefore, the precise anatomical localization of the electrodes on the patient’s brain plays a key role in the definition of the epileptogenic zone [6] or in the mapping of eloquent cortex [7]. From a clinical point of view, the accurate localization of the anatomical boundaries of the epileptogenic zone allows excluding eloquent areas, avoiding deficits to patient and minimizing brain volume resection. The localization of these electrodes is generally obtained by matching the locations of the electrodes with the brain anatomy of the patient [8].

Commonly, a pre-implant magnetic resonance image (MRI) is co-registered to a post-implant computed tomography scan (CT) [9, 10], because MRI offers higher brain tissue contrast, while CT supports electrodes localization [5], even if CT images are affected by metal artifacts.

Various dedicated software tools that support pre-surgical evaluation are currently available as MATLAB-based packages or open-source software, also with graphical user interfaces. They mainly provide MRI-CT co-registrations and offer only basic features for recognition of ECoG electrodes from CT scans. Synoptic Table 1 reports the most recent software tools, also outlining their main features and limitations for electrodes localization. Most software segments the electrodes via simple image thresholding and requires manual interaction to correct the data. Manual methods are very time-consuming, user-dependent and prone to inaccuracy. On the other hand, the mere CT image thresholding method is not able to recognize all the electrodes and to completely exclude other metal objects, such as wires, tooth fillings, intracranial clips, splinters, stitches, hearing aids and intracranial stents. Hence, manual intervention is often required to adjust the data. The ALICE tool, proposed in [11], considers the volume of segmented clusters to identify the electrodes, but turned out to be unable to exclude other objects with comparable volumes (e.g., wire clusters).

**Table 1:** List of software tools for epilepsy pre-surgical evaluation and their approach for electrodes recognition

Reference, name	Proposed approach	Limitations
[10]	Manual + threshold	It is time-consuming and operator-dependent
[6]	Threshold	Other metallic objects are not excluded
[8]	Threshold	It requires the neurosurgeon to previously estimate trajectories and target points
[12]	Threshold	It is not successful in the presence of nearby wires, skull artifacts or overlapping electrodes
[13, 14], iELVis, BioImage Suite	Manual + threshold	It is time-consuming and operator-dependent
[15], iElectrodes	Manual + threshold	It requires manual selection of electrode voxels and not-electrodes objects must be manually removed
[11], ALICE	Threshold + clustering	It requires manual selection of overlapping or missing electrodes
[16]	Threshold	It is time-consuming and a semi-manual identification of each electrode centroid must be performed by an expert user
[17], iEEGview	Threshold + manual	It requires manual electrodes identification to obtain their 3D coordinates

This paper presents a novel, more robust, automated method to recognize ECoG electrodes in CT volumes. It consists of identification of metal objects and analysis of their shapes to recognize ECoG electrodes among all detected objects and provide their locations. The proposed approach can be easily implemented in existing tools.

## Materials and methods

### Patients cohorts

#### Neuromed database

Head CT scans of 24 patients (10 females and 14 males, age 35.4 ± 9.25 years) undergoing epilepsy surgery were provided by the “IRCCS Neuromed” (Pozzilli, Italy) and included in this study. Before the acquisition of CT scans, patients underwent craniotomy and ECoG electrodes were placed onto their brain surface. CT images were acquired by a General Electric LightSpeed Pro 16 Multi-Slice scanner. RX tube parameters were set to 120 kV, 600 mA for a total of 37 mAs. The CT gantry was not tilted. The pixel size ranged from 0.44 to 0.98 mm, and the slice thickness was 0.625 mm.

**Fig. 1:**
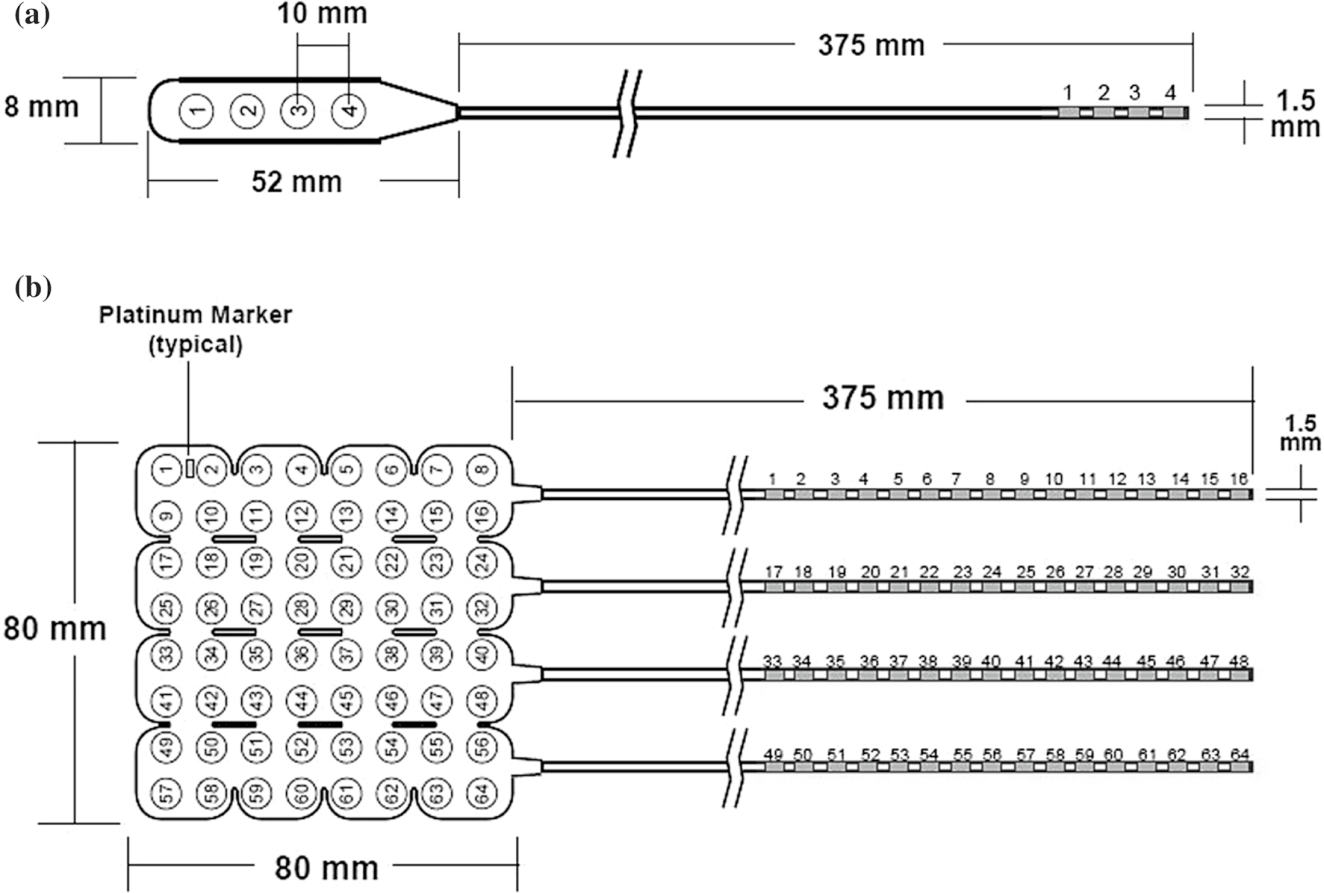
Sketch of the ECoG electrodes arrays: **a** Strip of 4 electrodes; **b** Grid of 8 × 8 electrodes (www.adtechmedical.com, Catalog #: IS04R-SP10X-000, Catalog #: FG64C-SP10X-0C6)

Flexible ECoG electrode arrays (Ad-Tech Medical Instrument Corporation, 400 West Oakview Parkway Oak Creek, WI 53,154 USA) were used. Each electrode consisted of a platinum–iridium disc with a diameter of 4 mm and a thickness of about 0.5 mm. Electrodes inter-distances were 10 mm (nominal center-to-center spacing). Electrode arrays were embedded in flexible sheets and arranged either in strips or grids (see Fig. 1). The strips contained 4 (Fig. 1a), 6, 8 or 12 electrodes, while the grids were composed of matrices of 8 × 8 (Fig. 1b) or 6 × 8 electrodes. They were conveniently placed where needed, onto the frontal, temporal or parietal cortex. Electrodes were connected to the recording device via CABRIO or TECH-ATTACH cables, ending with standard 1.5 mm safety socket DIN connectors. Grid electrodes enclosed a platinum marker to identify electrode numbered as 1 (see Fig. 1b). Table S1 (the current table and all the subsequent ones are available in supplementary materials) shows the number of implanted electrodes, strips, grids and other metal objects per patient.

**Fig. 2:**
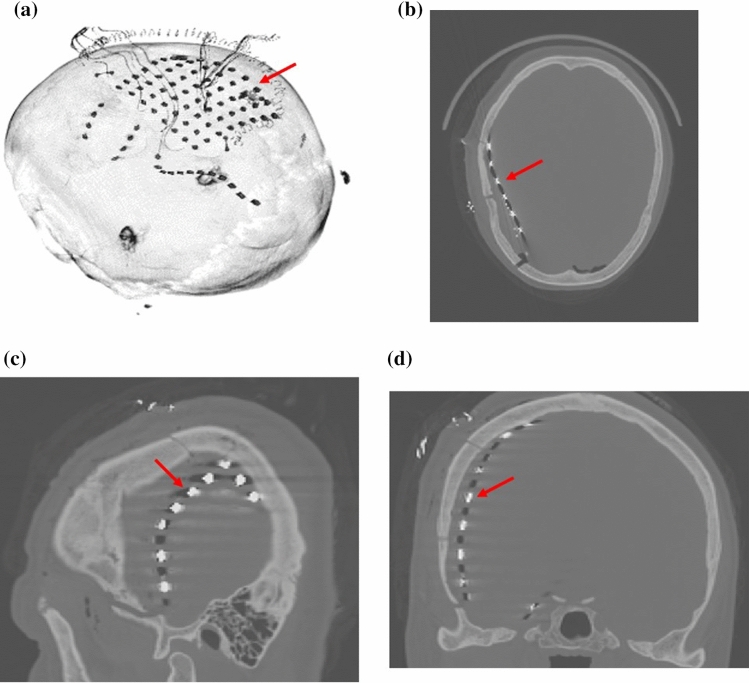
CT volume of Neuromed patient #15: **a** 3D rendering, **b–d** axial, sagittal and coronal cut planes. The red arrows point to electrodes. In the 3D rendering both an 8 × 8 grid electrodes and some strip electrodes are clearly visible. Cranium cuts are clearly recognizable in the axial view

As an example, Fig. 2 shows a 3D rendering (Fig. 2a) of the CT volume of Neuromed patient #15, along with the axial (Fig. 2b), sagittal (Fig. 2c) and coronal (Fig. 2d) cut planes. ECoG electrodes are indicated by red arrows.

#### Mayo database

A smaller patient cohort from a database of CT volumes, made available by Mayo Clinic (200 First St. SW Rochester, MN 55,905, USA) on the IEEG public repository [18], was also considered. In particular, only the studies feature a complete head volume and a resolution comparable to electrodes size. The CT volumes including only ECoG electrodes were first considered in the analysis, that is, the ones with Mayo patient IDs #12, #16, #20, #22, #26, #28, #31 (two females and five males, age 17.4 ± 13.8 years).

Moreover, the CT volumes with penetrating depth electrodes (arrays of coaxial sleeve-shaped electrodes arranged on a thin tip) were also considered, i.e., the ones with Mayo patient IDs #5, #17, #27 (one female and two males, age 33.0 ± 6.56 years). In particular, the first two include only depth electrodes, while the third includes both depth and ECoG electrodes. Table S2 reports the number of implanted ECoG electrodes, strips, grids, depth electrodes, depth contacts and other metal objects per patient.

### Electrodes recognition workflow

**Fig. 3:**
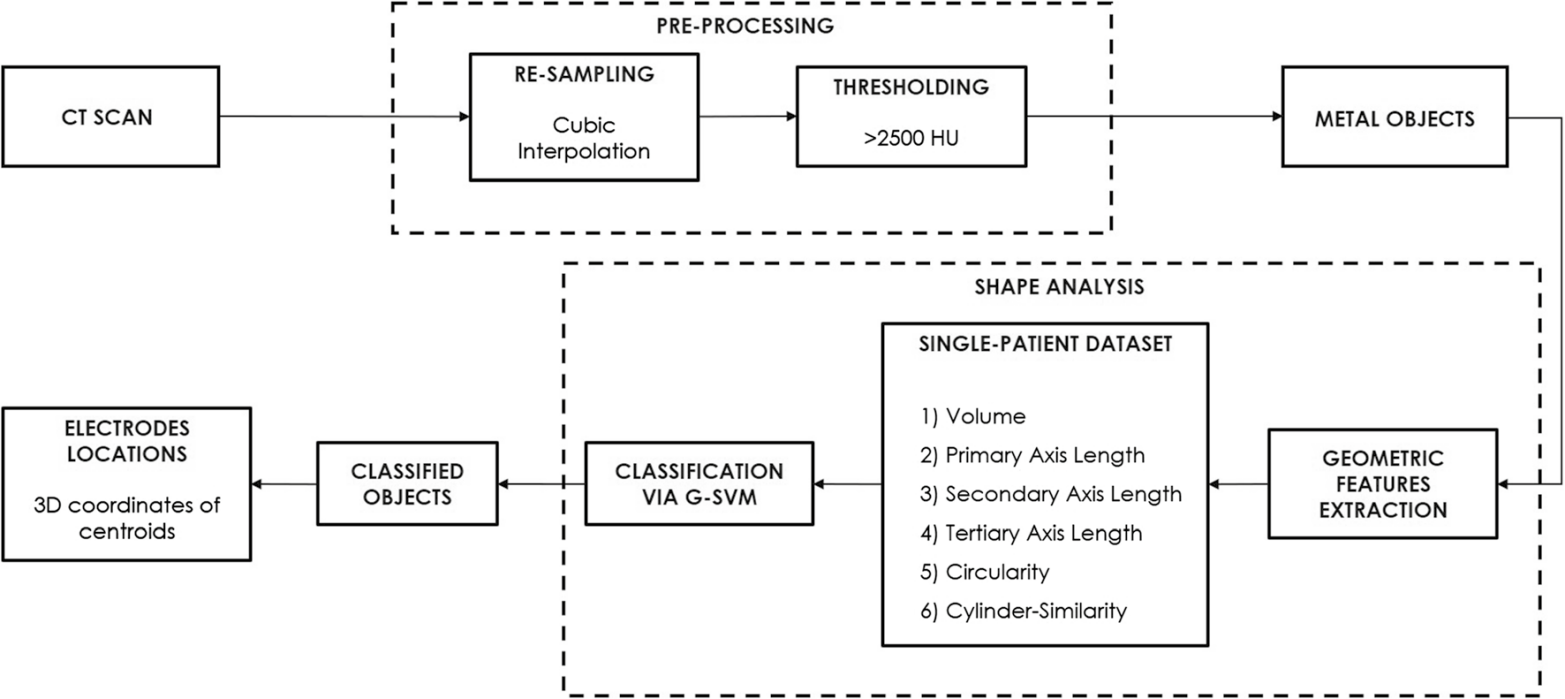
Workflow of the proposed automated method for electrodes recognition

Figure 3 shows the workflow of the proposed automated method for electrodes recognition. A first preprocessing stage is implemented to identify all the metal objects within the CT volume. Afterward, a shape analysis is performed to recognize the actual electrodes among all the metal objects previously detected. The subsequent paragraphs explain the steps of the proposed method in details. In this study, all the described steps were performed in MATLAB^®^.

#### CT pre-processing

The CT volumes were first re-sampled via a cubic interpolation, to obtain a cubic voxel of 0.5 mm side, which makes the geometric features rotationally invariant. Then, a thresholding on radiodensity values (Hounsfield Units, HU) was performed to detect the metal objects within the CT volume. As shown in Fig. 3, all metal objects with high attenuation coefficients were identified by using an HU threshold of 2500. (It is greater than the typical HU values of compact bone.) This operation identified the electrodes, but also wires, stitches, connectors, metal dental fillings. Therefore, the thresholding was not able to selectively identify the electrodes. A large part of the wires and stitches were located outside patients’ head.

#### Shape analysis

The binary volumes obtained after the thresholding were processed to identify clusters of six-connected voxels (i.e., with at least six faces attached to another voxel above the threshold). Then, a shape analysis of these binary clusters was carried out to separate the electrodes from the other metal objects. Six geometric features were extracted for each cluster of voxels: (1) Volume; (2) Primary axis length; (3) Secondary axis length; (4) Tertiary axis length; (5) Circularity and (6) Cylinder similarity. The volume, primary, secondary and tertiary axes lengths were computed via the MATLAB® function “regionprops3,” which also provided the 3D coordinates of centroids belonging to each cluster of voxels. The volume is defined as the number of voxels belonging to the cluster. The primary, secondary and tertiary axes lengths (sorted from the highest to the lowest) correspond to those of an ellipsoid that entirely comprises the cluster [19]. The circularity describes the roundness of a cluster and is defined as:

1 $$ Circularity = \frac{primary\, axis \,length}{{secondary \,axis \,length}} $$

The cylinder similarity indicates how similar the cluster is to a cylinder with a diameter equal to the average of primary and secondary axes lengths, and the height equal to the tertiary axis length. It is defined as:

2 $$ Cylinder - similarity = \frac{{\left( {\frac{primary \,axis\, length + secondary \,axis\, length}{4}} \right)^{2} \cdot \pi \cdot tertiary\, axis \,length}}{volume} $$

The electrodes essentially have the shape of a considerably flattened cylinder (like a small coin), therefore, they should have circularity and cylinder similarity both equal to 1; on the contrary, the circularity and cylinder similarity of threads and sutures segments, which have an elongated and potentially curved shape, should exhibit substantial deviations from unity.

The shape analysis is divided in two steps (see Fig. 3), namely the geometric features extraction and the classification. The former is aimed at extracting the considered geometric features for each of the metal objects within the CT volume, as well as their centroids, and organizing them in a proper dataset; the latter takes such dataset as input and provides the predicted class for all considered objects as output. A training phase is usually required for a classifier to achieve good performances and demands the a priori knowledge of the true class for each object. Indeed, this is required by the classifier to learn the optimal criteria for discriminating between instances of different classes.

In practice, before being able to use the proposed method to automatically recognize electrodes, the construction of a training dataset via feature extraction and manual classification, as well as the classifier training are mandatory. To this aim, a distinct dataset was built for each patient, with rows corresponding to all metal objects within the CT volume, and columns to the six geometric features and a manually assigned class. By considering the 24 Neuromed single-patient datasets, and the seven Mayo single-patient datasets including only ECoG electrodes (Mayo patient IDs #12, #16, #20, #22, #26, #28, #31), two classes were considered: “ECoG” and “Non-electrode”. Moreover, for the two Mayo single-patient datasets including only depth electrodes (Mayo patient IDs #5, #17), the two classes were: “Depth” and “Non-electrode.” Finally, in case of the Mayo single-patient dataset with both ECoG and depth electrodes (Mayo patient ID #27; 12 depth electrodes, 35 ECoG electrodes and 92 non-electrodes), three classes were taken into account: “ECoG”, “Depth” and “Non-electrode”. “ECoG” and “Depth” classes were assigned to the actual electrodes, while the “Non-electrode” class was assigned to all the other metal objects detected (screws, cables, etc.).

Furthermore, combined datasets were also built and named as:

- “C1” (1753 ECoG electrodes, 17928 non-electrodes) obtained by joining all Neuromed single-patient datasets;
- “C2” (531 ECoG electrodes and 4848 non-electrodes) obtained by joining the seven Mayo single-patient datasets containing ECoG electrodes (IDs #12, #16, #20, #22, #26, #28, #31);
- “C3” (32 depth electrodes, 531 ECoG electrodes and 5970 non-electrodes) obtained by joining the seven Mayo single-patient datasets containing ECoG electrodes (IDs #12, #16, #20, #22, #26, #28, #31) and the two Mayo single-patient datasets containing depth electrodes (IDs #5, #17).

A Gaussian support vector machine (G-SVM) [20] was used as a classifier to discriminate between the considered classes and its classification performances were assessed by applying the tenfold cross-validation on each single-patient and combined dataset. In tenfold cross-validation, the dataset is randomly divided into ten subsets of equal size, and then each subset is tested using the classifier trained on the remaining nine subsets. Then, the obtained ten classification accuracies are averaged to provide an overall classification accuracy [20].

Further analyses were carried out by using completely distinct datasets for classifier training and testing (i.e., without using the tenfold cross-validation on the same dataset). First, the feasibility of recognizing ECoG electrodes in CT volumes of a medical center by using a classifier trained on data acquired from another center was investigated. To this aim, a G-SVM classifier was trained on the combined dataset C1 and tested on the single-patient datasets with Mayo IDs #12, #16, #20, #22, #26, #28, #31. Finally, the three-class classifier trained on the combined dataset C3 was tested on the Mayo single-patient dataset ID #27.

## Results

### Descriptive statistics of geometric features on Neuromed database

**Fig. 4:**
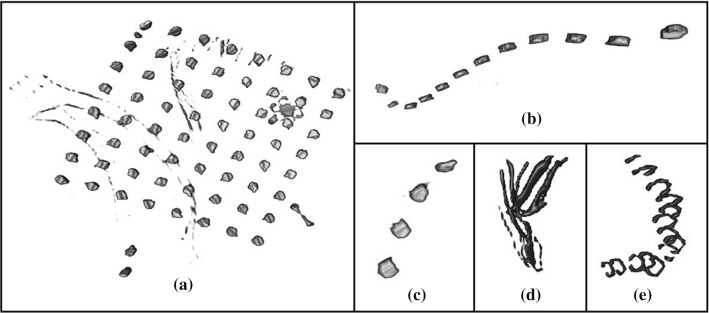
Renderings from the CT volume of Neuromed patient #15 after thresholding (HU > 2500): **a** 64 electrode grid, two more electrodes and other structures (e.g. wires); **b** 12 electrodes strip; **c** 4 electrodes strip; **d** bundle of electrode wires; **e** stitches or clips next to each other

As an example, Fig. 4 shows some details renderings from the CT volume of Neuromed patient #15, obtained after the thresholding operation. Figure 4a clearly shows a grid of 64 electrodes (note the platinum marker between the top electrode and the next lower left), other two electrodes below the grid, as well as some wires and stitches, which hinder simple automatic electrodes recognition. Figure 4b, c shows strips of 12 and 4 electrodes, respectively. Figure 4d clearly shows a bundle of some wires, while Fig. 4e shows various stitches.

**Fig. 5:**
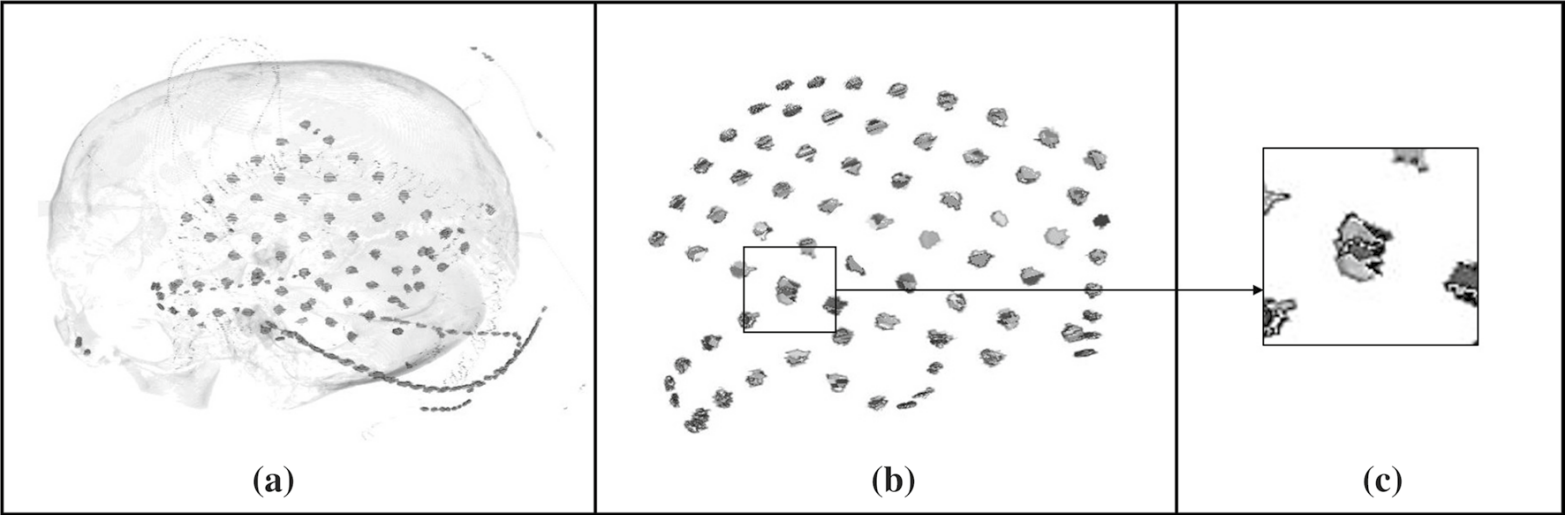
Example of overlapping electrodes (false negative): **a** 3D volume rendering of the CT volume of Neuromed patient #9; **b** The overlapping of one electrode belonging to a grid and another one belonging to a strip generates a unique cluster, classified as a single electrode; **c** Magnification of panel **b** showing the two overlapped electrodes

The following information was extracted to obtain statistics on voxels clusters that represent the electrodes. Each cluster actually corresponding to an electrode was manually selected (e.g., those represented in Fig. 4c) for a total number of 1753 electrodes. Thirteen electrodes turned out to be fused with other electrodes or structures and were discarded. In detail, for Neuromed patient #4, #5, #11, #22, two electrodes were partially placed one above the other; this overlap generated a unique, larger cluster comprising both electrodes. Also, in the case of Neuromed patient #9, one electrode belonging to a grid and another one belonging to a strip were superimposed (see Fig. 5).

Table S3 outlines the descriptive statistics (mean; standard deviation; minimum, 25^th^ percentile; median, 75^th^ percentile; maximum) of the six considered geometric features of ECoG and non-electrode classes.

**Fig. 6:**
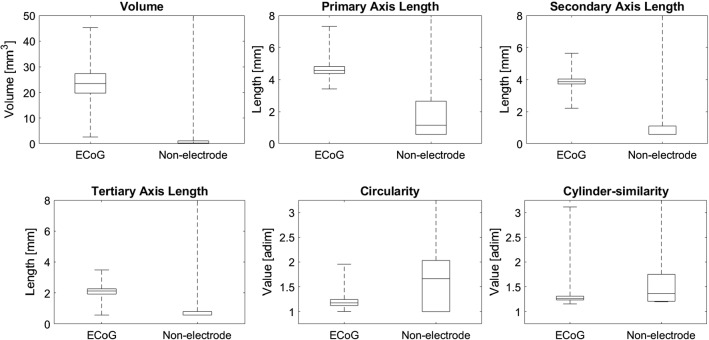
Box and whiskers plots of the geometric features of electrodes and non-electrodes. The continuous lines within the boxes indicate the median values, the lower and upper boxes limits indicate the 25th and 75th percentiles, and the whiskers lengths indicate the maximum and minimum values

Figure 6 depicts the compared box and whiskers plots of each of the ECoG and non-electrodes features, while Fig. S1 (available in the supplementary materials) shows the occurrence histograms of each of the six considered geometric features for the ECoG class.

### Performances of shape analysis for electrodes recognition

#### ECoG electrodes from Neuromed datasets

Table S4 shows the classification accuracies obtained by the G-SVM on each Neuromed single-patient dataset. The average classification accuracy across all patients was 99.74% (SD: 0.2967%), the related confusion matrix (right and wrong average recognition percentages across all 24 patients) is shown in Fig. 9a, and both the false negatives and false positives per patient are reported in Table S5.

The classification accuracy achieved by the G-SVM on the combined dataset C1 was 99.74% and the related confusion matrix (right and wrong average recognition percentages) is shown in Fig. 9b.

**Fig. 7:**
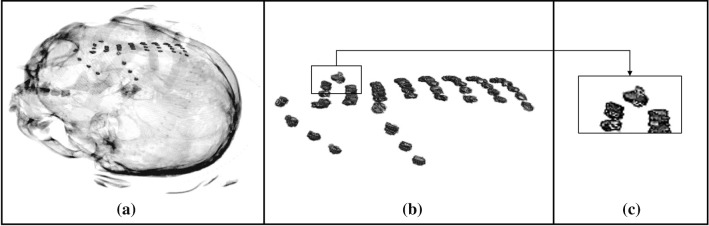
Example of a metallic agglomerate mistakenly classified as an electrode (false positive): **a** 3D volume rendering of the CT volume of Neuromed patient #21; **b** Metal objects detected in the image processing phase; **c** Magnification of panel **b** showing the metallic screw (the topmost element in the rectangle)

As an example, Fig. 7 shows the case of a metal agglomerate, made up of a metal screw, mistakenly classified as an electrode (false positive) in the Neuromed single-patient dataset #21.

Table S6 outlines the classification performances accomplished on the combined dataset C1, by using a G-SVM with different features combinations. The “volume” feature was considered as the most representative of an ECoG electrode, and for this reason was always taken into account. The highest classification accuracy (99.74%) was achieved on the combined dataset C1 by considering all features. It is worth noticing that all analyzed feature combinations achieved classification accuracies in excess of 99%, except for the classification based on the volume alone.

#### ECoG electrodes from Mayo clinic datasets

Table S7 shows the classification accuracies obtained by the G-SVM classifier on each of the seven Mayo single-patient datasets (Mayo patient IDs #12, #16, #20, #22, #26, #28, #31). The average classification accuracy across all patients was 98.27% (SD: 1.971%), the related confusion matrix is shown in Fig. 9c, and both the false negatives and false positives per patient are reported in Table S8.

The G-SVM achieved a classification accuracy of 99.68% on the combined dataset C2, and the related confusion matrix is shown in Fig. 9d.

Table S9 outlines the classification performances achieved on the combined dataset C2 by considering different features combinations. The highest classification accuracy (99.68%) was achieved on the combined dataset C2 by considering all features. All analyzed features combinations achieved classification accuracies in excess of 99%, except for the classification based on the volume alone.

**Fig. 8:**
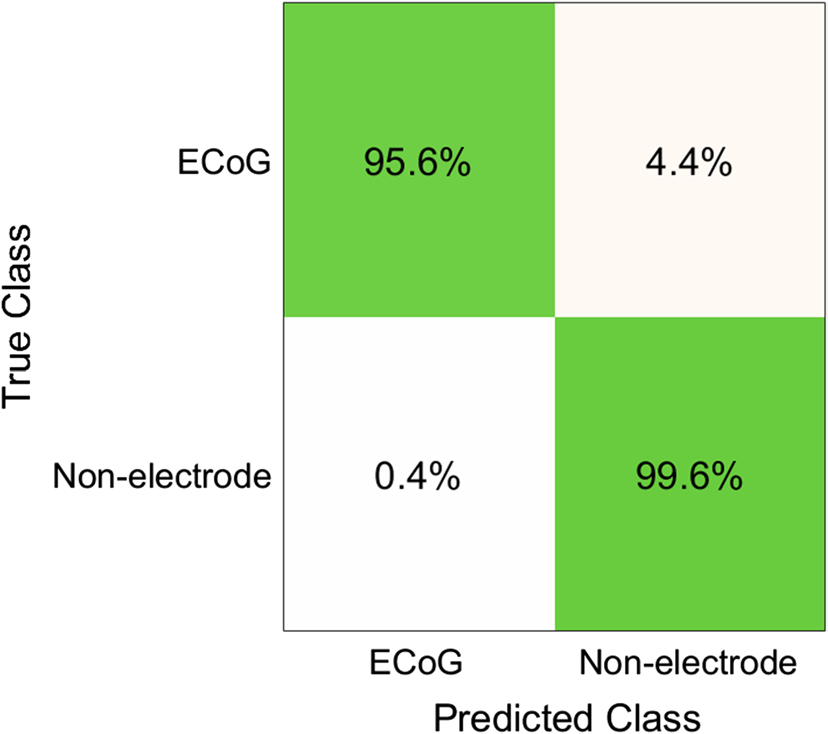
Confusion matrix, averaged across all patients, presenting the classification accuracies (in percentages) achieved on the datasets with Mayo patient IDs #12, #16, #20, #22, #26, #28, #31, by using a G-SVM classifier trained on the combined dataset C1 (Neuromed database). Rows correspond to true classes and columns to predicted classes

Table S10 outlines the classification performances achieved by testing, on the seven Mayo single-patient datasets (IDs #12, #16, #20, #22, #26, #28, #31), a G-SVM classifier that had been previously trained on the combined dataset C1. The average classification accuracy across all patients was 98.94% (SD: 0.9932%), and the related confusion matrix is shown in Fig. 8.

#### Depth and ECoG electrodes from Mayo datasets

**Fig. 9:**
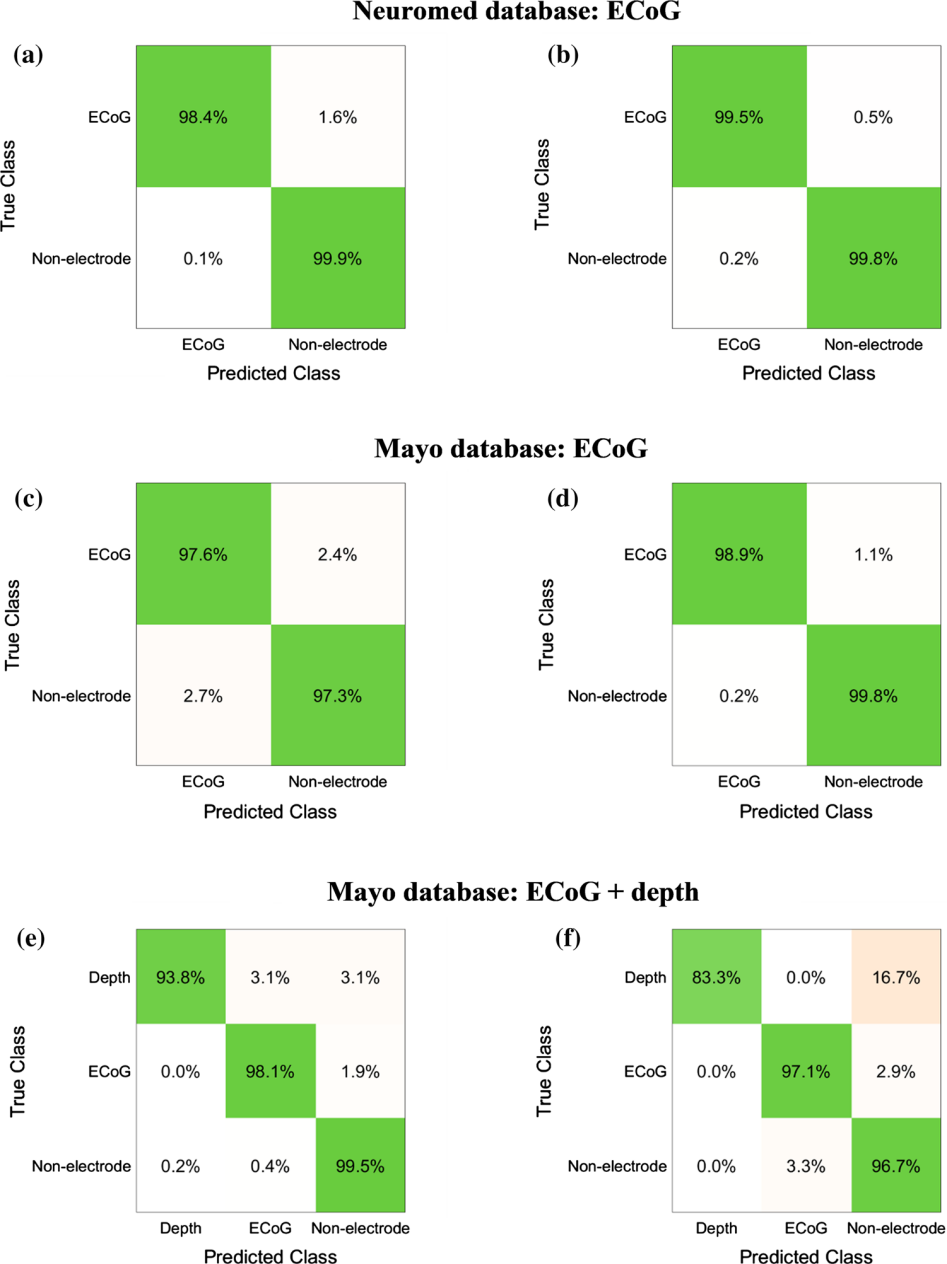
Confusion matrices presenting the G-SVM classification accuracies: **a** averaged across all Neuromed single-patient datasets; **b** computed on the combined dataset C1(Neuromed database); **c** averaged across on the seven Mayo single-patient datasets with only ECoG electrodes; **d** computed on the combined dataset C2 (Mayo database); **e** computed on the combined dataset C3 (Mayo database); **f** computed on the Mayo single-patient dataset #27, with the G-SVM trained on the combined dataset C3

The first test for depth and ECoG electrodes recognition was carried out on the combined dataset C3. The three-class G-SVM achieved an accuracy of 99.34%, as well as a sensitivity of 93.75% and 97.93% and a specificity of 99.86% and 99.63%, for depth and ECoG electrodes, respectively. The related confusion matrix is shown in Fig. 9e.

A final test was carried out on the Mayo single-patient dataset #27, which had not been used for the previous classifier training. The G-SVM classifier, previously trained on the combined dataset C3, achieved an overall accuracy of 95.68%, as well as a sensitivity of 83.33% and 97.14%, and a specificity of 100.0% and 97.06%, for depth and ECoG electrodes, respectively. The related confusion matrix is shown in Fig. 9f.

## Discussion and conclusions

This study focused on the specific task of automated ECoG electrodes recognition from CT volumes. Currently, only basic automated algorithms are available for this task, which are based on thresholding methods. Indeed, electrodes exhibit higher radiodensities than compact bone, which facilitates their detection by applying a proper threshold on HU values. However, other metal objects (e.g., stiches, clips, connecting wires of electrodes) exhibit such high HU values too, thus impairing electrodes recognition. Moreover, it is well-known that any metal object causes streak metal artifacts on CT images, which may cause alterations of electrodes shapes. Hence, these methods still require intensive, time-consuming manual intervention, to obtain a reasonable accuracy in electrodes localization.

The method presented in this article addresses these issues by means of an automated shape analysis based on machine learning. In particular, the method extracts geometric features of metal objects and applies G-SVM classification to identify the disc-shaped electrodes. It is worth mentioning that the Neuromed database does not include any CT scan acquired with tilted gantry, nor cases with micro-electrodes and penetrating depth electrodes. However, the performances of the proposed method were further assessed on CT volumes from a public repository, which included also penetrating depth electrodes.

The analysis of the results highlighted that the partial volume effect caused an increase in the volume of the electrodes determined from the CT volumes, as compared to their real size. Indeed, the volume of voxel clusters corresponding to an electrode resulted more than double of its real value. This is very evident from the mismatch between the actual electrodes thickness and the length of the tertiary axis. Furthermore, the spatial orientation of electrodes with respect to the CT slice planes caused slight alterations of their shape, which may explain the variability observed in the geometric features. The problem of overlapping electrodes remains unsolved and still requires manual intervention or the adoption of suitable strategies [11]. In addition, metal artifacts could imply issues in the correct recognition of electrodes with very small inter-distances, since they could be detected as superimposed.

The results obtained both on the 24 Neuromed single-patient datasets and on the seven Mayo single-patient datasets (IDs #12, #16, #20, #22, #26, #28, #31) show a very high percentage of recognized ECoG electrodes with rejection of almost all the other metal objects. The G-SVM average classification accuracies across all patients were 99.74% and 98.27% for the Neuromed and Mayo databases, respectively. The G-SVM achieved comparable performances also on the combined datasets C1 and C2, by scoring classification accuracies of 99.74% and 99.68%, respectively. The higher accuracy accomplished on the combined dataset C2 from the Mayo database, as compared to the related mean accuracy across patients, was reasonably due to the availability of more extensive information on ECoG electrodes features, which led to a more efficient classifier training. Remarkable results were also achieved on the combined datasets by considering a lower number of features, which resulted in classification accuracies in excess of 99%, apart from using the volume alone.

Moreover, the performances of a G-SVM classifier, trained on the combined dataset C1 (Neuromed database), were assessed by testing it on the combined dataset C2 (Mayo database), which had not been used for classifier training. In these tests, the G-SVM scored a mean classification accuracy of 98.94%, which was higher than the one obtained by the tenfold cross-validation performed on the same seven Mayo single-patient datasets. These results confirm, as expected, that the classifier performances benefited from training on a larger dataset, but they also suggest the possibility to apply our method also without training on new data, by using a previously trained classifier. It should be underlined that the limited availability of data for Mayo patients with only ECoG electrodes did not allow to train the classifier on a number of instances comparable to those of Neuromed datasets.

Further tests were carried out on the three additional Mayo single-patient datasets (IDs #5, #17, #27), which also included depth electrodes. A three-class G-SVM classifier was used to separately recognize depth and ECoG electrodes from all other metal objects. The results of the tenfold cross-validation on the combined dataset C3 show a surprisingly high overall percentage of correctly classified objects (~ 99%), with sensitivities to depth and ECoG electrodes, respectively, of 93.75% and 97.93%, and specificities of 99.86% and 99.63%. The three-class G-SVM classifier, previously trained on the combined dataset C3, was tested on the Mayo single-patient dataset #27, which had never been used for classifier training. The classifier scored an overall accuracy of about 96%, with sensitivities to depth and ECoG electrodes, of 83.33% and 97.14%, and specificities of 100.0% and 97.06%. The apparent reduction in the sensitivity to depth electrodes was mainly due to the very small number of instances included in the test set. The encouraging results obtained in depth electrodes recognition by using the same geometric features considered for ECoG electrodes could be ascribed to the CT finite resolution and the partial volume effect, which probably transformed the sleeve-shaped electrodes in full cylinder-like solids. However, the number of depth electrodes the method has been tested on is not as statistically relevant as that of ECoG electrodes in the Neuromed database. Therefore, a more extensive investigation should be carried out in future studies to assess the actual performances of the proposed method for depth electrodes recognition.

The proposed automated method, even when trained with a very limited training set, is able to identify at least the 98% of ECoG electrodes in a CT scan with the 2.7% of misclassified electrodes. When properly trained on a sufficient number of instances, it is able to recognize more than 99% of ECoG electrodes with less than 1% of misclassified electrodes. Hence, to the best of our knowledge, the proposed method achieves unprecedented recognition accuracy, and could provide a substantial reduction in the effort and time consumption required for manual intervention. Moreover, the method proved capable of recognizing depth and ECoG electrodes simultaneously in the same CT volume, thus it could be used also in recent studies that involve both electrodes types. However, in order to attain the highest classification performances, a proper classifier training should be performed which requires the availability of a sufficient number of instances related to electrodes with comparable shape, size and arrangement. Indeed, the use of very few data and/or data obtained with different electrodes can limit the performance of the classifier. Finally, the proposed method can be easily implemented into software suites, such as iELVis [13] and ALICE [11], which are widely used to manage the whole preoperative analysis process.

## Supplementary Information

Below is the link to the electronic supplementary material.

Supplementary file1 (DOCX 112 KB)

## Data Availability

The code that supports the findings of this study is available on GitHub at https://github.com/Jcentracchio/Automated-localization-of-ECoG-electrodes-in-CT-volumes.

## Data availability

Because of the sensitive nature of the data, it is available upon request to the corresponding author.
