# Supplementary material for: Efficient automated localization of ECoG electrodes in CT images via shape analysis
Source: Int J Comput Assist Radiol Surg. 2021 Mar 9;16(4):543–54. doi: 10.1007/s11548-021-02325-0 (PMC8052236; doi:10.1007/s11548-021-02325-0)
Supplement: Supplementary file 1 — Supplementary file1 (DOCX 112 KB) [file 11548_2021_2325_MOESM1_ESM.docx]

**INTERNATIONAL JOURNAL OF COMPUTER ASSISTED RADIOLOGY AND SURGERY**

**Efficient Automated Localization of ECoG Electrodes in CT Images Via Shape Analysis**

Jessica Centracchio^1,*^, Antonio Sarno^2^, Daniele Esposito^1,3^, Emilio Andreozzi^1,3^, Luigi Pavone^4^, Giancarlo Di Gennaro^4^, Marcello Bartolo^4^, Vincenzo Esposito^4,5^, Roberta Morace^4^, Sara Casciato^4^, Paolo Bifulco^1,3^

^1^ Department of Electrical Engineering and Information Technologies, Polytechnic and Basic Sciences School, University of Naples Federico II, Naples, Italy; [jessica.centracchio@unina.it](mailto:jessica.centracchio@unina.it); [daniele.esposito@unina.it](mailto:daniele.esposito@unina.it); [emilio.andreozzi@unina.it](mailto:emilio.andreozzi@unina.it); [paolo.bifulco@unina.it](mailto:paolo.bifulco@unina.it)

^2^ National Institute for Nuclear Physics (INFN), Naples, Italy; [sarno@na.infn.it](mailto:sarno@na.infn.it)

^3^ Department of Neurorehabilitation, IRCCS Istituti Clinici Scientifici Maugeri, Pavia, Italy

^4^ IRCCS Neuromed, Pozzilli, Italy; [bioingegneria@neuromed.it](mailto:bioingegneria@neuromed.it); [g.digennaro@neuromed.it](mailto:g.digennaro@neuromed.it); [bartolonrx@gmail.com](mailto:bartolonrx@gmail.com); [roberta.morace@yahoo.it](mailto:roberta.morace@yahoo.it); [sara_casciato@hotmail.com](mailto:sara_casciato@hotmail.com)

^5^ Department of Human Neurosciences, Sapienza University, Rome, Italy; [vincenzo.esposito@uniroma1.it](mailto:vincenzo.esposito@uniroma1.it)

* corresponding author: [jessica.centracchio@unina.it](mailto:jessica.centracchio@unina.it)

***Table S1*** *Number of implanted electrodes, strips, grids and other metal objects for each patient from Neuromed database*

| **Neuromed Patient ID** | **Number of ECoG electrodes** | **Number of strips** | **Number of grids** | **Number of other metal objects ^a^** |
| --- | --- | --- | --- | --- |
| #1 | 106 | 7 | 1 | 478 |
| #2 | 48 | 0 | 1 | 270 |
| #3 | 64 | 0 | 1 | 375 |
| #4 | 84 | 3 | 1 | 5622 |
| #5 | 98 | 5 | 1 | 960 |
| #6 | 48 | 0 | 1 | 406 |
| #7 | 48 | 0 | 1 | 235 |
| #8 | 48 | 0 | 1 | 289 |
| #9 | 76 | 4 | 1 | 941 |
| #10 | 78 | 5 | 1 | 1153 |
| #11 | 79 | 4 | 1 | 238 |
| #12 | 69 | 4 | 1 | 1194 |
| #13 | 48 | 0 | 1 | 657 |
| #14 | 88 | 5 | 1 | 341 |
| #15 | 84 | 3 | 1 | 619 |
| #16 | 60 | 1 | 1 | 245 |
| #17 | 86 | 3 | 1 | 312 |
| #18 | 82 | 3 | 1 | 476 |
| #19 | 80 | 2 | 1 | 335 |
| #20 | 94 | 5 | 1 | 954 |
| #21 | 56 | 1 | 1 | 323 |
| #22 | 86 | 3 | 1 | 407 |
| #23 | 59 | 3 | 1 | 384 |
| #24 | 98 | 6 | 1 | 714 |

^a^ Non-electrode objects identified after the thresholding on HU values

***Table S2*** *Number of implanted ECoG electrodes, strips, grids, depth electrodes, depth contacts and other metal objects for each patient from Mayo database*

| **Mayo Patient ID** | **Number of ECoG electrodes** | **Number of strips** | **Number of grids** | **Number of depth electrodes** | **Number of depth contacts** | **Number of other metal objects ^a^** |
| --- | --- | --- | --- | --- | --- | --- |
| #5 | 0 | 0 | 0 | 2 | 16 | 265 |
| #12 | 84 | 3 | 2 | 0 | 0 | 2542 |
| #16 | 64 | 4 | 2 | 0 | 0 | 298 |
| #17 | 0 | 0 | 0 | 2 | 16 | 857 |
| #20 | 56 | 4 | 2 | 0 | 0 | 49 |
| #22 | 60 | 2 | 2 | 0 | 0 | 45 |
| #26 | 96 | 5 | 1 | 0 | 0 | 181 |
| #27 | 36 | 3 | 1 | 3 | 12 | 92 |
| #28 | 96 | 2 | 2 | 0 | 0 | 629 |
| #31 | 116 | 5 | 2 | 0 | 0 | 1104 |

^a^ Non-electrode objects identified after the thresholding on HU values

***Table S3*** *Descriptive statistics of the geometric features of ECoG and Non-electrodes classes of the combined dataset C1 (Neuromed database)*

| **Features** | **Class** | **Mean** | **SD** | **Min** | **25^th^ perc.** | **Median** | **75^th^ perc.** | **Max** |
| --- | --- | --- | --- | --- | --- | --- | --- | --- |
| **Volume [mm^3^]** | Electrode | 23.57 | 5.232 | 2.625 | 19.75 | 23.5 | 27.38 | 45.25 |
|  | Non-Electrode | 4.552 | 41.71 | 0.1250 | 0.1250 | 0.2500 | 1.125 | 2047 |
| **Primary axis length [mm]** | Electrode | 4.64 | 0.396 | 3.43 | 4.39 | 4.58 | 4.82 | 7.32 |
|  | Non-Electrode | 2.80 | 6.45 | 0.577 | 0.577 | 1.155 | 2.660 | 223.1 |
| **Secondary axis length [mm]** | Electrode | 3.89 | 0.254 | 2.22 | 3.74 | 3.88 | 4.04 | 5.65 |
|  | Non-Electrode | 1.19 | 1.98 | 0.577 | 0.577 | 0.577 | 1.11 | 86.0 |
| **Tertiary axis length [mm]** | Electrode | 2.10 | 0.295 | 0.58 | 1.93 | 2.12 | 2.26 | 3.48 |
|  | Non-Electrode | 0.789 | 0.808 | 0.577 | 0.577 | 0.577 | 0.808 | 72.9 |
| **Circularity [adim]** | Electrode | 1.20 | 0.119 | 1.00 | 1.12 | 1.18 | 1.25 | 1.96 |
|  | Non-Electrode | 1.98 | 1.61 | 1.00 | 1.00 | 1.66 | 2.03 | 30.0 |
| **Cylinder-similarity [adim]** | Electrode | 1.29 | 0.104 | 1.16 | 1.24 | 1.26 | 1.31 | 3.11 |
|  | Non-Electrode | 2.20 | 7.87 | 1.20 | 1.21 | 1.36 | 1.75 | 714 |

*
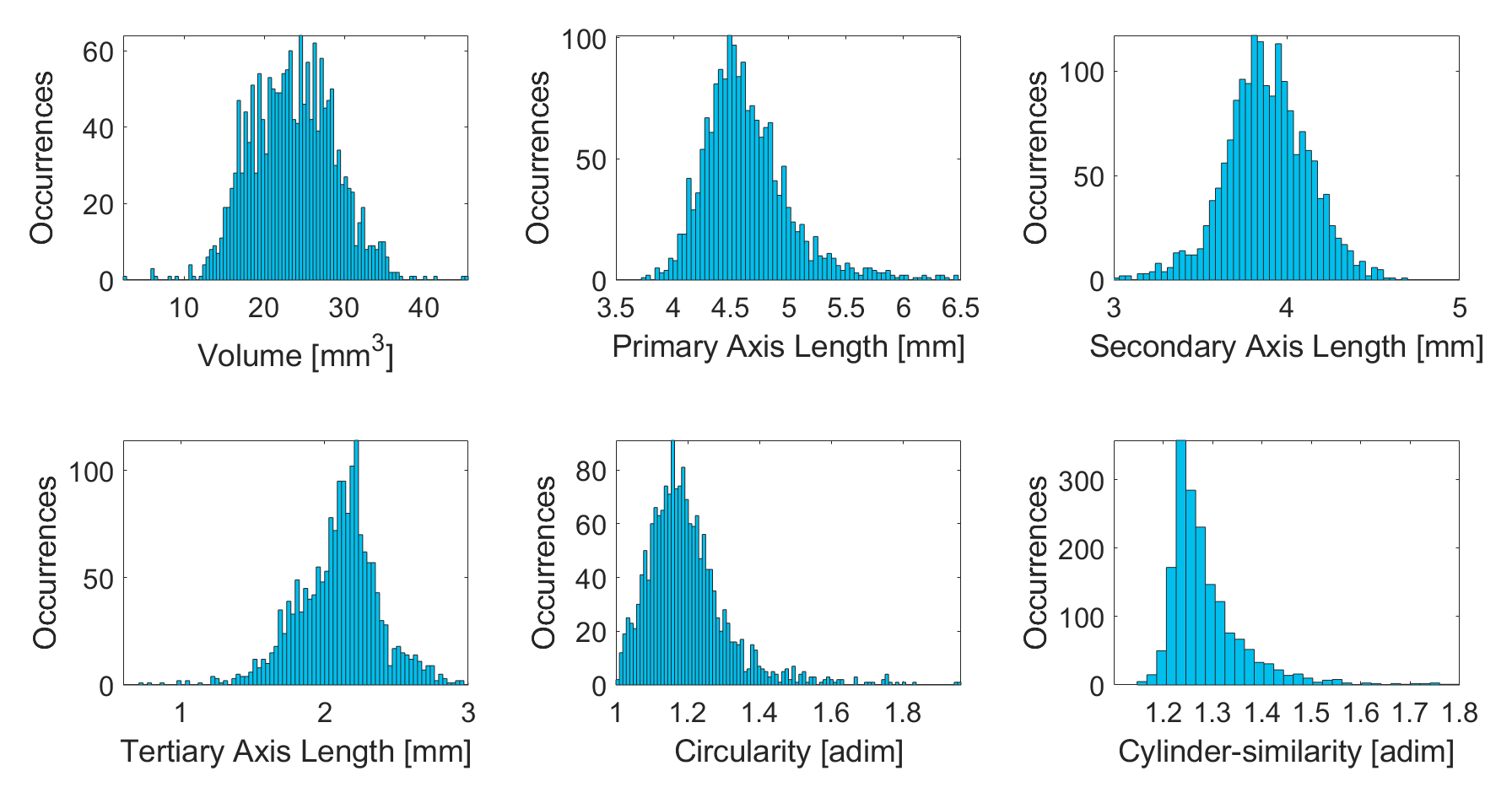
*

***Fig. S1*** *Occurrence histograms of geometric features for the ECoG class (computed for Neuromed patients)*

***Table S4*** *G-SVM classification accuracies (in percentage) of ECoG electrodes recognition on 24 distinct single-patient datasets*

| **Neuromed Patient ID** | **Classification accuracy (%)** |
| --- | --- |
| #1 | 99.83 |
| #2 | 100.0 |
| #3 | 100.0 |
| #4 | 99.81 |
| #5 | 100.0 |
| #6 | 99.78 |
| #7 | 98.94 |
| #8 | 100.0 |
| #9 | 99.70 |
| #10 | 99.76 |
| #11 | 100.0 |
| #12 | 99.60 |
| #13 | 99.72 |
| #14 | 99.77 |
| #15 | 99.43 |
| #16 | 100.0 |
| #17 | 99.50 |
| #18 | 99.46 |
| #19 | 99.04 |
| #20 | 99.62 |
| #21 | 99.74 |
| #22 | 100.0 |
| #23 | 100.0 |
| #24 | 100.0 |

**Table S5** False negatives and false positives of ECoG electrodes recognition per patient from the results of the 10-fold cross-validation (Neuromed)

| **Neuromed Patient ID** | **False negatives**  **(missed electrodes)** | **False positives**  **(misclassified electrodes)** |
| --- | --- | --- |
| #1 | 4 | 0 |
| #2 | 0 | 0 |
| #3 | 1 | 0 |
| #4 | 0 | 0 |
| #5 | 2 | 0 |
| #6 | 2 | 1 |
| #7 | 0 | 0 |
| #8 | 2 | 2 |
| #9 | 5 | 6 |
| #10 | 0 | 0 |
| #11 | 1 | 3 |
| #12 | 0 | 1 |
| #13 | 1 | 0 |
| #14 | 0 | 3 |
| #15 | 0 | 0 |
| #16 | 0 | 0 |
| #17 | 2 | 1 |
| #18 | 3 | 0 |
| #19 | 0 | 0 |
| #20 | 0 | 0 |
| #21 | 0 | 0 |
| #22 | 4 | 1 |
| #23 | 1 | 1 |
| #24 | 1 | 0 |

***Table S6*** *Classification accuracies accomplished on the combined dataset C1 by using a G-SVM with different features combinations (Neuromed database)*

| **Selected feature/s on combined database** | **Classification accuracy (%)** |
| --- | --- |
| volume | 98.48 |
| volume and circularity | 99.45 |
| volume and cylinder-similarity | 99.58 |
| volume and primary, secondary, tertiary axes lengths | 99.72 |
| volume; primary, secondary, tertiary axes lengths**;** circularity**;** cylinder-similarity | 99.74 |

***Table S7*** *Classification accuracies of ECoG electrodes recognition achieved by the G-SVM on datasets from Mayo Clinic database*

| **Mayo Patient ID** | **Classification accuracy (%)** |
| --- | --- |
| #12 | 99.81 |
| #16 | 98.86 |
| #20 | 97.09 |
| #22 | 94.29 |
| #26 | 99.28 |
| #28 | 98.89 |
| #31 | 99.67 |

**Table S8** False negatives and false positives of ECoG electrodes recognition per patient from the results of the 10-fold cross-validation (Mayo database)

| **Mayo**  **Patient ID** | **False negatives**  **(missed electrodes)** | **False positives**  **(misclassified electrodes)** |
| --- | --- | --- |
| #12 | 3 | 2 |
| #16 | 0 | 4 |
| #20 | 0 | 3 |
| #22 | 1 | 5 |
| #26 | 1 | 1 |
| #28 | 8 | 0 |
| #31 | 0 | 4 |

***Table S9*** *Classification accuracies accomplished on the combined dataset C2 from the Mayo database by using a G-SVM with different features combinations*

| **Selected feature/s on combined database** | **Classification accuracy (%)** |
| --- | --- |
| volume | 98.68 |
| volume and circularity | 99.49 |
| volume and cylinder-similarity | 99.41 |
| volume and primary, secondary, tertiary axes lengths | 99.63 |
| volume; primary, secondary, tertiary axes lengths**;** circularity**;** cylinder-similarity | 99.68 |

***Table S10*** *Classification accuracies of ECoG electrodes recognition achieved on datasets from Mayo Clinic database by using a G-SVM trained on the combined dataset C1 from Neuromed database*

| **Mayo Patient ID** | **Classification accuracy (%)** |
| --- | --- |
| #12 | 99.70 |
| #16 | 98.29 |
| #20 | 99.03 |
| #22 | 97.14 |
| #26 | 100.0 |
| #28 | 98.75 |
| #31 | 99.67 |
